# Do advertisements for antihypertensive drugs in Australia promote quality prescribing? A cross-sectional study

**DOI:** 10.1186/1471-2458-8-167

**Published:** 2008-05-20

**Authors:** Brett D Montgomery, Peter R Mansfield, Geoffrey K Spurling, Alison M Ward

**Affiliations:** 1 Discipline of General Practice, School of Primary, Aboriginal and Rural Health Care, University of Western Australia, Claremont, Western Australia, Australia; 2 Discipline of General Practice, University of Adelaide, Adelaide, South Australia, Australia; 3 Healthy Skepticism, Willunga, South Australia, Australia; 4 Discipline of General Practice, University of Queensland, Herston, Queensland, Australia; 5 Department of Primary Health Care, University of Oxford, Headington, Oxford, UK

## Abstract

**Background:**

Antihypertensive medications are widely prescribed by doctors and heavily promoted by the pharmaceutical industry. Despite strong evidence of the effectiveness and cost-effectiveness of thiazide diuretics, trends in both promotion and prescription of antihypertensive drugs favour newer, less cost-effective agents. Observational evidence shows correlations between exposure to pharmaceutical promotion and less ideal prescribing. Our study therefore aimed to determine whether print advertisements for antihypertensive medications promote quality prescribing in hypertension.

**Methods:**

We performed a cross-sectional study of 113 advertisements for antihypertensive drugs from 4 general practice-oriented Australian medical publications in 2004. Advertisements were evaluated using a quality checklist based on a review of hypertension management guidelines. Main outcome measures included: frequency with which antihypertensive classes were advertised, promotion of thiazide class drugs as first line agents, use of statistical claims in advertisements, mention of harms and prices in the advertisements, promotion of assessment and treatment of cardiovascular risk, promotion of lifestyle modification, and targeting of particular patient subgroups.

**Results:**

Thiazides were the most frequently advertised drug class (48.7% of advertisements), but were largely promoted in combination preparations. The only thiazide advertised as a single agent was the most expensive, indapamide. No advertisement specifically promoted any thiazide as a better first-line drug. Statistics in the advertisements tended to be expressed in relative rather than absolute terms. Drug costs were often reported, but without cost comparisons between drugs. Adverse effects were usually reported but largely confined to the advertisements' small print. Other than mentioning drug interactions with alcohol and salt, no advertisements promoted lifestyle modification. Few advertisements (2.7%) promoted the assessment of cardiovascular risk.

**Conclusion:**

Print advertisements for antihypertensive medications in Australia provide some, but not all, of the key messages required for guideline-concordant care. These results have implications for the regulation of drug advertising and the continuing education of doctors.

## Background

Hypertension is a major risk factor for cardiovascular disease [1] and the most common single problem managed in Australian general practice. [2] For more than a decade expensive new antihypertensive drugs have been prescribed more frequently than the older and more cost effective thiazide diuretics. [3-6] Newer antihypertensive drugs are among the highest volume and cost items for the Australian Pharmaceutical Benefits Scheme (PBS). [7] It is estimated that adherence to guidelines favouring older, less expensive agents in 1998 in Australia may have saved $45 to $108 million. [3] These guidelines promote antihypertensive drugs to augment lifestyle change, but not as a substitute for such change. [1,8,9]

The content of print advertising for antihypertensive drugs mirrors the trends away from cost-effective prescribing. [10] There is observational evidence of associations between doctors' prescribing and doctors' exposure to advertisements. [11-16] Thus, heavy promotion may be at least partly responsible for the more frequent prescribing of newer drugs. Indeed, recognition that marketing influences prescribing has prompted government-funded social marketing campaigns to encourage more cost-effective choices. [17] Doctors may not be aware of the degree to which their prescribing is influenced by advertising. [18]

The Medicines Australia Code of Conduct states that all promotional information "must be current, accurate, balanced and must not mislead either directly, by implication, or by omission". [19] The Australian National Medicines Policy (NMP) states that each partner (including the medicines industry) "accepts that all must be engaged in a cooperative endeavour to bring about better health outcomes for all Australians, focusing especially on people's access to, and wise use of, medicines." The NMP definition of "quality use of medicines" includes taking into account "the potential risks and benefits of treatment, dosage, length of treatment, and cost."[20] We believe evidence-based guidelines are a useful standard for evaluating how well advertisements support, rather than lead away from, wise use of medicine. Thus, the aim of our study was to determine whether print advertisements for antihypertensive medications in Australia promote prescribing for hypertension that is concordant with evidence-based guidelines. To answer this, we reviewed evidence-based guidelines for important prescribing messages, and then looked for these messages in a sample of advertisements from Australian medical publications.

At the time of performing our study, we were unaware of any other similar studies regarding messages in advertisements for antihypertensive medicines. Since then a study of antihypertensive drug advertising in a Dutch journal has been published. This study found 35% of the advertisements contained claims unsupported by evidence. [21] Our research builds on previous studies of advertisements and promotional brochures which have found overemphasis on relative statistical measures[22,23], and claims lacking clarity, accuracy, balance and substantiation. [22-25]

## Methods

In the course of our literature review we identified several major international hypertension guidelines from the World Health Organisation, the US and Europe,[1,8,26] as well as several Australian guidelines and prescribing aids[9,27-29] and other recent publications. [30-34] These were all current and relevant at the time we sampled our advertisements. The guidelines agreed on several key messages for the effective treatment of hypertension and quality use of medicines (table 1). However they were not entirely consistent on one important issue: the choice of first- line drug. Although all guidelines endorsed thiazides as a first-line drug class, some listed other classes as equally first-line whilst some promoted thiazides specifically as the first-line class in the absence of compelling indications for another class. This difference between guidelines may arise from different interpretations of the ALLHAT[31] and ANBP2 trials,[35] and from differing emphasis on the importance of cost-effectiveness. Given that the ALLHAT trial had in our opinion a stronger design, and that the NMP definition of quality use of medicines includes consideration of costs, we regarded the recommendation of thiazides as the first-line class to be correct.

**Table 1:** Key messages in evidence-based hypertension management

• Modification of lifestyle. [1, 8, 9, 26-29, 32, 34]
• Assessment of overall cardiovascular risk. [1, 9, 26-29, 32, 34]
• Reduction of overall cardiovascular risk. [1, 8, 9, 26-29, 32, 34]
• Patients at low to medium risk may be given a longer trial of lifestyle changes before commencing pharmacotherapy. [9, 26-29, 32, 34]
• A range of drug treatments exists, including diuretics, beta-blockers, angiotensin-converting enzyme (ACE) inhibitors, calcium channel blockers, and angiotensin receptor antagonists. [1, 8, 9, 26-29, 32, 34]
• The choice of antihypertensive drug may depend on characteristics of the patient, including other medical conditions or use of other medications. [1, 8, 9, 26-29, 32, 34]
• In the absence of compelling indications for a different agent, start drug treatment with a low-dose thiazide. [1, 8, 27-33]
• Thiazides are more cost-effective than newer agents in the management of hypertension. [1, 3, 30, 31]
• If a patient requires a combination of agents in the treatment of blood pressure, a low dose thiazide should usually form part of the combination. [1, 8, 29, 32]

Using these key messages from hypertension management guidelines, we developed a quality checklist to extract data from the advertisements (see additional file 1: checklist.pdf). The checklist was used to measure promotion of lifestyle modification, promotion of thiazides as first line agents, promotion of cardiovascular risk assessment, promotion of use of the drug for specific subgroups of patients, and the mention of alternative antihypertensive classes as treatment options (these issues are all drawn from the quality prescribing messages in table 1). We regarded advertisements as not fully promoting guideline-concordant care if they did not mention all of the items on the check list. We also counted the frequency of mention of drug harms and drug prices, because those factors are included in the NMP definition of quality use of medicines. Finally, we assessed statistical claims, because use of relative rather than absolute measures may have a stronger influence on doctors' prescribing,[36,37] and previous research has demonstrated a lack of reporting of absolute measures in pharmaceutical advertisements and brochures. [22,23]

We sampled advertisements for antihypertensive drugs from four advertisement-rich, general practice-oriented Australian medical publications. We drew these from issues published between February and July of 2004, inclusive. We examined every issue of the monthly publications (*Australian Family Physician *and *Medicine Today*) and one randomly selected issue per month of each of the weekly publications (*Australian Doctor *and *Medical Observer*). The random selection was based on randomly-generated integers from a web-based random number generator. [38] Our analysis included the largest advertisement (by page area) for each antihypertensive drug in each issue. Where two or more equally sized advertisements for the same drug existed, both or all were included. We excluded "short" advertisements (as defined by the Medicines Australia Code of Conduct). [19]

We evaluated text and graphs in the advertisements, but not imagery (thus, pictures of sports shoes, people swimming, and so on were not counted as promotion of physical activity). We examined each entire advertisement including contiguous "fine print", but we did not assess further information on other pages of the journals. (Only six advertisements in our sample of 113 referred readers to such non-contiguous fine print.) For some items, we stratified our findings according to whether information was present in the "main body" of the advertisement; for these purposes we excluded the "fine print" and "PBS Information" sections of the advertisement (see additional file 1: checklist.pdf).

Each advertisement was rated independently by two authors, with disagreements resolved by consensus. By designing a checklist which was largely "yes or no" in available responses, we tried to minimise subjective interpretative difficulties. Inter-rater reliability was not formally calculated. The few inter-rater differences we found were usually due to oversights, and consensus was easily achieved. Descriptive results in the form of counts and frequencies were calculated using Microsoft Excel.

In our paper, we use the term "thiazide class drug" to refer to traditional thiazides (bendrofluazide and hydrochlorothiazide) as well as the other "thiazide-like" low-ceiling diuretics chlorthalidone and indapamide. Guidelines tend to recommend these interchangeably, and meta-analysis evidence suggests they have equivalent effects. [39]

## Results

113 advertisements met our inclusion criteria. These were composed of 27 unique advertisement designs which appeared between 1 and 14 times each.

### Antihypertensive drug classes

Frequencies of advertisements for members of different antihypertensive drug classes are shown in Table 2. More than one drug class was advertised in 41 (36.3%) advertisements; of these, 28 advertised multiple products, and 34 advertised combination medicines.

**Table 2:** Categorisation of 113 advertisements by class(es) of the antihypertensive drug(s) promoted

Drug Class	Advertisements which advertised drug from this drug class N (%*)	Advertisements mentioning this drug class, or member of class, in main body text of advertisement N (%*)	Advertisements mentioning this drug class, or member of class, in entire advertisement, including fine print N (%*)
Thiazide diuretics	55 (48.7%)	64 (56.6%)	85 (75.2%)
Beta-blockers	0 (0%)	9 (8.0%)	16 (14.2%)
ACE inhibitors	44 (38.9%)	49 (43.4%)	53 (46.9%)
Angiotensin receptor blockers	29 (25.7%)	31 (27.4%)	31 (27.4%)
Calcium antagonists	26 (23.0%)	26 (23.0%)	26 (23.0%)

* The numerator for the percentages is the 113 advertisements included in the study. Percentages add to more than 100% because many advertisements mentioned more than one drug class.

Of the 55 advertisements for a thiazide class drug, 21 were for indapamide as a single-agent pill. The remaining 34 were for combination pills, of which 14 were for indapamide-containing preparations, and 20 were for hydrochlorothiazide-containing preparations (see table 3). We found no advertisements for the only other thiazide class drugs available in Australia at the time of our study: chlorthalidone or bendrofluazide.

**Table 3:** Categorisation of the 55 thiazide advertisements by product(s) promoted

Thiazide advertised as:	Thiazide advertised	
	
	indapamide N (%*)	hydrochlorothiazide N (%*)
Single agent thiazide pill	18 (15.9%)	0 (0%)
Single agent thiazide pill plus single agent ACE inhibitor pill	3 (2.7%)	0 (0%)
Combination thiazide/ACE inhibitor pill	0 (0%)	0 (0%)
Combination thiazide/angiotensin receptor blocker pill	0 (0%)	13 (11.5%)
Combination thiazide/ACE inhibitor pill plus single agent ACE inhibitor pill	14 (12.4%)	6 (5.3%)
Combination thiazide/angiotensin receptor blocker pill plus single agent angiotensin receptor blocker pill	0 (0%)	1 (0.9%)
Totals	35 (31%)	20 (17.7%)

* The numerator for the percentages is the 113 advertisements included in the study.

No advertisements unequivocally stated that, in the absence of compelling indications for a different agent, drug treatment should start with a low-dose thiazide. The closest example to this statement, which we found in two advertisements, was: "diuretics are suitable first-line agents – but be wary of the long term effects of secondary hyperglycaemia, hyperuricaemia, renal dysfunction and electrolyte imbalance, particularly in the elderly". [40]

Seventy-three (64.6%) advertisements specifically reminded the reader to consider antihypertensive medications other than the advertised agent(s). Fifteen advertisements advised consideration of another drug instead of the advertised agent; the other 58 advertisements advised consideration of the other drug in addition to the advertised agent.

### Use of statistics

Efficacy statistics were included in 33 (29.2%) advertisements. Three of these clearly stated that these statistics were relative risk reductions. No advertisements presented statistics in the form of absolute risk reductions, numbers needed to treat or harm, or survival benefit. Seven advertisements used risk reduction statistics without specifying whether they were absolute or relative. These seven advertisements contained nine different risk reduction claims. Examination of the advertisements' references revealed that all of these nine statistics were relative risk reductions or estimates thereof derived from odds ratios. 23 advertisements included statistical claims about surrogate endpoints (for example, average reduction in blood pressure or proportion of treated patients who reached a target blood pressure).

Statistical significance was mentioned in 7 (6.2%) advertisements. Of these, 4 stated p-values. No advertisements clearly reported confidence intervals. Three advertisements mentioned a 95% confidence interval without providing the interval boundaries or stating whether or not the results were actually statistically significant.

### Other issues

Possible harm from taking the advertised drug was mentioned in 99 (87.6%) advertisements. In all but 2 of the advertisements, this mention of harm was confined entirely to the fine print.

Most advertisements (104 (92%)) mentioned prices, and all of these described the PBS dispensed price for the medicine. Fifteen (13.3%) advertisements mentioned that there was no PBS brand price premium for the advertised agent. No advertisements explicitly compared the cost effectiveness of different drug classes.

With regards to lifestyle interventions, no advertisements promoted smoking cessation, weight loss, or exercise. Alcohol and salt were the only dietary interventions discussed. Restricting alcohol intake was advised in 53 (46.9%) advertisements, but this advice was always in the small print in the context of a potential interaction with the advertised drug. Sodium restriction was mentioned in 54 (47.8%) advertisements, and was always confined to small print warnings of potential adverse effects if the advertised agents were given to a salt-restricted patient. This contrasts with hypertension guidelines, which advise decreasing dietary sodium as a means of reducing hypertension. No advertisements specifically advised trialling lifestyle changes before commencing drug treatment.

Forty-six (40.7%) advertisements promoted their drug for a particular subgroup of patients in the main body text (for details, see additional file 2: subgroups.pdf). Of these, fifteen (13.3%) promoted their drug for a high-risk population such as patients with existing macrovascular disease or diabetic patients with hypertension.

Seventy-five (66.4%) advertisements claimed that their drug reduced risk, danger or harm. Forty-seven (41.6%) advertisements specifically characterised these risks as cardiovascular or cerebrovascular events (angina, MI, TIA, CVA or similar). Three (2.7%) advertisements suggested the assessment, measurement or estimation of the patient's overall cardiovascular risk. Excluding comments on drug interactions, 3 (2.7%) advertisements recommended consideration be given to pharmacological treatments to lower cardiovascular risk other than antihypertensive drugs. These were aspirin and lipid-lowering agents. Optimisation of diabetic control with medication was not promoted.

## Discussion and conclusion

To our question of whether Australian antihypertensive advertisements promote quality prescribing, our results offer a mixed answer, demonstrating some important missed opportunities for the promotion of quality use of antihypertensives.

We found that messages to prescribers about lifestyle interventions were almost absent from the advertisements. This bias towards promotion of drugs rather than lifestyle change is problematic because lifestyle interventions should be first line treatment for many hypertensive patients, and the foundation of ongoing treatment even when medications are used.

Much information that is important for quality care was underemphasised or not provided by the advertisements. PBS costs of medications were frequently reported in small print but cost comparisons within and between drug classes were missing. In most advertisements, information about harms was confined to the fine print. There was a lack of emphasis on assessing and modifying patients' overall cardiovascular risk.

The promotion of medications for specific target groups may help the individualisation of therapy for the patient in an evidence-based manner. However, advertising claims based on data from higher-risk patients may artificially heighten a prescriber's sense of a drug's efficacy for patients at lower risk. Similarly, the over-emphasis on relative rather than absolute statistics may create over-optimistic impressions of efficacy and thus encourage over-prescribing. [36,37] Also, if doctors and patients perceive drugs as very effective, then they may overlook the need for lifestyle change.

The absence of advertisements for beta-blockers may be helpful, given that recent meta-analyses show less favourable outcomes for beta-blockers (particularly atenolol) than other antihypertensive classes. [41,42] (While these meta-analyses were not published at the time of our sampling of advertisements, most of the primary trials on which they were based were published.)

In our sample, thiazides were the most advertised class, contrasting strongly with a previous study which found that thiazide advertising in the New England Journal of Medicine declined from 4.2% of advertising pages in 1985 to 0% in 1996. [10] In our study, the most frequently advertised thiazide was indapamide. This drug is priced almost twice as high as some other equally effective thiazides. The only other thiazide advertised was hydrochlorothiazide, which was only advertised in combination medications. Clearly, the advertisements do not encourage the prescribing of the most cost-effective drugs as a first-line monotherapy.

Our study has several limitations. The validity of our results may be limited by the number of advertisements surveyed. The generalisability of our results is uncertain because we focused only on advertisements in Australia during 2004. We did not examine the role of imagery and emotional appeals in the advertisements, because it is difficult to assess these objectively. We did not attempt to assess the accuracy of therapeutic claims made in the advertisements. We also did not attempt, for those advertisements promoting antihypertensive agents for particular patient subgroups, to determine whether a particular class of antihypertensive would have been the most appropriate choice for that particular subgroup (in fact, such issues are often contested in the literature).

We used evidence-based guidelines as a gold standard for evaluating advertisements. The industry may advocate acceptance of lower standards but we believe higher standards for pharmaceutical promotion are required by the Australian NMP.

Guidelines may not be perfect. Sometimes recommendations vary between guidelines. The guidelines selected for our study varied in their recommendations about first-line drugs so our decision to favour thiazides is open to discussion. Guidelines may be biased by vested interests including drug companies. [43]

Our study describes the content of the advertisements, but is not designed to show association or causation between exposure to promotion and prescribing behaviour. Our study should be read, though, in the context of existing observational evidence of such associations. [11-15,44-46]

Despite these limitations, we believe our results indicate that many Australian print advertisements for antihypertensive medications lack elements important for cost-effective care consistent with evidence-based guidelines. Our findings lend support to calls for increased availability of independent prescribing information, reform of the incentives for, and regulation of, pharmaceutical advertising,[47] and avoidance of promotional information by prescribers. [48] These results also support calls for medical publications to be helped to develop alternative business models rather than relying on drug promotion. [49]

## Competing interests

PRM is Director of Healthy Skepticism, an organisation which aims to improve health by reducing harm from misleading drug promotion. BDM and GKS are members of Healthy Skepticism. AMW declares that she has no competing interests.

## Authors' contributions

BDM, PRM and AMW conceived, designed and coordinated the study. BDM and PRM conducted the literature review and designed the quality checklist, with which they and GKS extracted data from the advertisements. BDM analysed the data and drafted the manuscript, to which all authors made important contributions. All authors read and approved the final manuscript.

## Pre-publication history

The pre-publication history for this paper can be accessed here:



## Supplementary Material

Additional file 1 The quality checklist used to extract data from the advertisements. Click here for file

Additional file 2 An additional table with details of promotion of antihypertensives for particular subgroups of patients. Click here for file
