# Supplementary material for: Do advertisements for antihypertensive drugs in Australia promote quality prescribing? A cross-sectional study
Source: BMC Public Health. 2008 May 20;8:167. doi: 10.1186/1471-2458-8-167 (PMC2409327; doi:10.1186/1471-2458-8-167)
Supplement: Additional file 1 — The quality checklist used to extract data from the advertisements. [file 1471-2458-8-167-S1.pdf]

Advertisement evaluation form

Who is rating this ad?

Advertisement Identification Number:

Questions in black type should always be answered, but questions in blue should be answered only if relevant according to the introductory question, and otherwise left blank.

Drug class

For the following drug classes, indicate whether this advertisement advertises a drug from the given class:

Thiazide or thiazide-like diuretic	Y	N
Beta blocker	Y	N
ACE inhibitor	Y	N
Angiotensin receptor antagonist	Y	N
Calcium channel blocker	Y	N
Other (specify if yes)	Y	N

If two or more were chosen, is this an advertisement for:

More than one antihypertensive preparation	Y	N
A combination preparation	Y	N

Statistics

Are efficacy statistics presented in this advertisement?	Y	N
If so, are statistics presented in terms of the following?		
Relative risk reduction	Y	N
Absolute risk reduction	Y	N
Numbers needed to treat	Y	N
Numbers needed to harm	Y	N
Survival benefit	Y	N
Other (specify)	Y	N
Non-specific (i.e. can’t tell from ad, would need to consult reference to find out)	Y	N

Are measures of statistical significance present in the advertisement?	Y	N
If so, are statistics presented in terms of the following?		
p value(s)	Y	N
Confidence interval(s)	Y	N
Text only – such as “statistically significant”	Y	N
Other (specify)	Y	N

Harms

Does the advertisement make any mention of possible harm to health as a consequence of taking the antihypertensive medication(s)?	Y	N
Are these harms only mentioned in the fine print?	Y	N

Cost

Does the advertisement make any mention of medication costs? Y N
Does the advertisement state the PBS dispensed price for the medication(s)? Y N
Does the advertisement give any information on costs or cost-effectiveness beyond stating the PBS dispensed price for the medication(s)? Y N
Does the advertisement give cost comparisons between this agent and agents of different classes? Y N
Does the advertisement give cost comparisons between this agent and agents from the same class? Y N

Statements about hypertension

Lifestyle factors

Are the following lifestyle factors mentioned in the text (main body or fine print) of the advertisement?

	Main body only	Entire ad, including fine print
Weight loss	Y N	Y N
Exercise	Y N	Y N
Dietary salt restriction	Y N	Y N
Other dietary modification	Y N	Y N
Alcohol use moderation	Y N	Y N
Smoking cessation	Y N	Y N
"Lifestyle" factors, unspecified	Y N	Y N
Other (specify)	Y N	Y N
If yes to alcohol above, then is alcohol only mentioned in terms of a possible interaction with the drug advertised?	Y N	Y N
If yes to salt above, then is salt only mentioned in terms of a possible adverse effect of prescribing the drug to a salt or sodium depleted patient?	Y N	Y N
Is there advice to first trial lifestyle changes (in patients at low or moderate overall cardiovascular risk, or in any patients) before beginning drug treatment?	Y N	Y N

Characteristics of the patient

Does the advertisement promote the drug or state the drug is indicated for "high blood pressure", "hypertension", or "essential hypertension" without qualification as to other patient characteristics? Y N
Does the text in the main body of the advertisement promote the drug for a particular subset of patients? Y N
If so, then in which of the following specific subgroups:
Secondary prevention of coronary artery disease Y N

Hypertension plus coronary artery disease Y N
Secondary prevention of cerebrovascular disease Y N
Hypertension plus cerebrovascular disease Y N
Isolated systolic hypertension Y N
Diabetes Y N
Diabetes plus hypertension Y N
Diabetes plus one additional cardiovascular risk factor (as per HOPE study criteria) Y N
Prevention of progression of renal failure in patients with proteinuria Y N
Patients uncontrolled on monotherapy Y N
Other (specify if yes) Y N

Cardiovascular risk

Does the advertisement make claims that its product reduces risk or danger or harm? Y N

If so, then are these risks or dangers or harms specifically characterised as cardiovascular events (angina, MI, TIA, CVA or similar)? Y N

Does the advertisement explicitly suggest or promote the assessment, measurement or estimation of the patient's overall cardiovascular risk? Y N

Other than comments on drug interactions, is any recommendation given to considering pharmacological treatments to lower cardiovascular risk other than antihypertensive drugs? Y N

If so, which treatments:
Aspirin Y N
Lipid-lowering therapy Y N
Treatment of diabetes Y N
Other (specify if yes) Y N

Range of Treatments

Which of the following drug classes are mentioned (or have one or more members of the class mentioned) in the main body text of the advertisement?

Thiazides Y N
Beta-blockers Y N
ACE inhibitors Y N
A2Ras Y N
CCBs Y N

Which of the following drug classes are mentioned (or have one or more members of the class mentioned) anywhere in the advertisement, including fine print?

Thiazides Y N
Beta-blockers Y N
ACE inhibitors Y N
A2Ras Y N
CCBs Y N

Does the advertisement specifically remind the prescriber to consider medications other than

the advertised agent(s)? Y N

If so, does it suggest the other medications in addition to or instead of the advertised agent?

additional instead both

Thiazide issues

Does the advertisement specifically state that, in the absence of compelling indications for a different agent, drug treatment should start with a low-dose thiazide? Y N

If the advertisement promotes a thiazide or thiazide-like agent, does it promote it as a single-agent preparation (i.e. not a combination pill)? Y N

If so, then which agent is the advertisement promoting?

Bendrofluazide Y N

Hydrochlorothiazide Y N

Chlorthalidone Y N

Indapamide Y N

Other (specify if yes) Y N

If promoting a combination therapy for hypertension, does the combination include a thiazide or thiazide-like agent? Y N

If so, is this:

Bendrofluazide Y N

Hydrochlorothiazide Y N

Chlorthalidone Y N

Indapamide Y N

Other (specify if yes) Y N
