# Supplementary material for: Do advertisements for antihypertensive drugs in Australia promote quality prescribing? A cross-sectional study
Source: BMC Public Health. 2008 May 20;8:167. doi: 10.1186/1471-2458-8-167 (PMC2409327; doi:10.1186/1471-2458-8-167)
Supplement: Additional file 2 — An additional table with details of promotion of antihypertensives for particular subgroups of patients. [file 1471-2458-8-167-S2.pdf]

Promotion of antihypertensives for particular subgroups of patients
(in main body of advertisement)

**Subgroup of patients**	**Number of advertisements** (% of total)
Isolated systolic hypertension	20 (17.7%)
Patients uncontrolled on monotherapy	12 (10.6%)
Diabetes plus hypertension	11 (9.7%)
Diabetes	5 (4.4%)
Secondary prevention of coronary artery disease	4 (3.5%)
Hypertension plus coronary artery disease	4 (3.5%)
Secondary prevention of cerebrovascular disease	3 (2.7%)
Hypertension plus cerebrovascular disease	3 (2.7%)
Diabetes plus one additional cardiovascular risk factor (as per HOPE study* criteria)	1 (0.9%)
Prevention of progression of renal failure in patients with proteinuria	0 (0%)
Other†	9 (8.0%)

* The Heart Outcomes Prevention Evaluation Investigators. Effects of an angiotensin-converting-enzyme inhibitor, ramipril, on cardiovascular events in high-risk patients. *N Engl J Med.* 2000; 342: 145–153.

† included asthma, renal impairment, heart failure, elderly patients and intermittently non-compliant patients.
